# Fermented rice bran supplementation mitigates metabolic syndrome in stroke-prone spontaneously hypertensive rats

**DOI:** 10.1186/s12906-016-1427-z

**Published:** 2016-11-08

**Authors:** Md Alauddin, Hitoshi Shirakawa, Takuya Koseki, Naoko Kijima, Ardiansyah, Slamet Budijanto, Jahidul Islam, Tomoko Goto, Michio Komai

**Affiliations:** 1 Laboratory of Nutrition, Department of Science of Food Function and Health, Graduate School of Agricultural Science, Tohoku University, 1-1 Tsutsumidori-Amamiyamachi, Aoba-ku, Sendai, 981-8555 Japan; 2 Faculty of Agriculture, Yamagata University, Tsuruoka, Japan; 3 Yamagata General Agricultural Research Center, Yamagata, Japan; 4 Department of Food Science & Technology, Universitas Bakrie, Jakarta, Indonesia; 5 Faculty of Food Technology, Bogor Agricultural University, Bogor, Indonesia

**Keywords:** Fermented rice bran, Metabolic syndrome, Adiponectin, Glucose tolerance, Angiotensin-converting enzyme, Stroke-prone spontaneously hypertensive rat

## Abstract

**Background:**

Previous study shown that enzyme treated-rice bran effectively improved hypertension and glucose intolerance in stroke-prone spontaneously hypertensive rat (SHRSP). However, dual fermentation of rice bran’s efficacy against metabolic syndrome in SHRSP is still unknown.

**Methods:**

Fermented rice bran (FRB) was prepared by dual fermentation of rice bran using fungi and lactic acid bacteria. The effect of FRB on metabolic syndrome in stroke-prone spontaneously hypertensive rats (SHRSP) was investigated by single and chronic supplementation.

**Results:**

Dual fermentation of rice bran enriches the functional value of rice bran. Single-dose oral administration of FRB (2 g/kg body weight) reduced systolic blood pressure; however, chronic supplementation with 5 % FRB (4 weeks) significantly reduced both systolic and diastolic blood pressure. FRB supplementation improved leptin impairment and increased serum adiponectin levels and angiotensin-converting enzyme inhibitory activity. Furthermore, FRB supplementation improved glucose tolerance and insulin sensitivity as well as serum insulin levels. Lipid profiles were also improved by the regulation of 5′ adenosine monophosphate-activated protein kinase activation. Moreover, supplementation with FRB reduced the expressions of hepatic transcription factors such as liver X receptor alpha, sterol regulatory element-binding protein 1c, and carbohydrate-responsive element-binding protein alpha, as well as their target genes. In conclusion, dietary supplementation with FRB may lower hypertension and alleviate metabolic syndrome.

**Conclusion:**

Metabolic syndrome was better alleviated with FRB supplementation. We therefore suggest FRB as an alternative medicine to reduce the risks of lifestyle-related diseases.

## Background

Metabolic syndrome is a multifactorial metabolic disorder characterized by hypertension, insulin resistance, dyslipidemia, and impaired glucose homeostasis. Approximately 25.0 % of the adult population worldwide has been diagnosed with metabolic syndrome. A recent meta-analysis showed that metabolic syndrome is associated with a 2-fold increase in cardiovascular outcomes [1, 2]. Several functional food candidates such as certain beverages, fruits, vegetables, grains, legumes, herbs, and spices are considered to prevent or moderate the lethal effects of metabolic syndrome [3]. Therefore, new therapeutic approaches are required to intensively lower the healthcare cost and better control the conditions associated with metabolic syndrome.

Rice bran is a byproduct of the rice milling process and a rich source of dietary fibre and various bioactive compounds [4]. Dietary fibre and phenolic compounds from rice bran are widely used to increase the functionalities of some foodstuffs and enhance their effects against chronic diseases [5]. Supplementation with rice bran and its active components has been shown to be effective against dyslipidemia in different animal models [6–8]. Thus, it is possible to derive an array of value-added products from rice bran for health promotion. This is because several active compounds such as oryzanols, tocopherols, tocotrienols, phytosterols, and nucleotides have been identified in rice bran [9–11]. Enzyme-treated rice bran has been shown to be effective against chronic diseases in rat and mouse models [12, 13]. For instance, a recent study showed that protein hydrolysates from rice bran improved insulin resistance in a mouse model [14]. Emerging evidence of the chronic disease-fighting properties of rice bran has advanced the development of rice bran for human use as a functional food and a dietary supplement [15].

Several techniques such as fermentation have been used in biotechnological applications to enhance the nutritional and functional values of rice bran to make the latter more beneficial in the management of metabolic syndrome. Previous studies have shown that rice bran fermented with *Saccharomyces cerevisiae* has antistress and antifatigue effects [16]. Furthermore, polysaccharides extracted from rice bran have shown anticancer and antidefective effects by regulating immune responses, whereas a water extract of fermented rice bran has shown an antiphotoaging effect [17, 18]. Ferulic acid and phenolic compounds isolated from rice bran have been shown to have hypoglycemic effects in a mouse model of type 2 diabetes [19]. Moreover, brown rice fermented with *Aspergillus oryzae* can suppress sodium dextran sulfate-induced colitis [20]. It has been shown in other studies that rice bran fractions improve glucose and lipid metabolism in animal models of metabolic syndrome [21, 22]. However, the effects of fermented rice bran (FRB) in metabolic syndrome have been examined in only a few studies.

In the present study, we prepared FRB, which has a higher nutritional value and flavour and is more palatable than non-fermented rice bran (Non-FRB), and estimated its metabolic effects in stroke-prone spontaneously hypertensive rats (SHRSP).

## Methods

### Materials

AIN-93M standard diet components were purchased from Wako Pure Chemical Industries, Ltd. (Osaka, Japan) and Oriental Yeast Co., Ltd. (Tokyo, Japan). An angiotensin-converting enzyme (ACE) inhibitory assay kit (ACE kit-WST; Dojindo, Kumamoto, Japan) and an insulin assay kit (Rat insulin ELISA kit; Morinaga Institute of Biological Science, Inc., Yokohama, Japan) were used in this study. Blood pressure (BP) was measured using the tail-cuff method (without heating) with a BP meter (MK-2000; Muromachi Kikai, Tokyo, Japan). Unless otherwise specified, all the chemicals and solvents used were of analytical grade. Rice bran was kindly provided from Sunbran Company (Tendo, Japan).

### Preparation of FRB

FRB was prepared by dual fermentation using fungi and lactic acid bacteria. At the first stage, rice bran was steamed and cooled to about 30 °C, after which a spore solution of *Aspergillus kawachii* was inoculated at an initial concentration of 10^6^ spores/g of rice bran. The rice bran was incubated at 30 °C in a fermentation chamber for 44 h. The solid-state culture obtained was designated as rice bran koji. A mixture of rice bran koji and rice powder (2:1) was saccharified using a 4-fold amount of water at 56 °C for 12 h, heated at 85 °C for 15 min, and then cooled to about 30 °C. At the second stage, the saccharified culture solution was inoculated with a mixture of lactic acid bacteria (*Lactobacillus brevis*, *Lactobacillus rhamnosus*, and *Enterococcus faecium*) at a concentration of 0.01 % (*w/w*). The solution was then incubated at 37 °C overnight and then heated at 85 °C for 15 min to obtain the FRB. The FRB solution was then filtered and lyophilized. The lyophilized powder was kept at −30 °C until use. Non-FRB was prepared by the same procedure mentioned above without the inoculation of the rice bran with fungi or lactic acid bacteria. The macronutrients in FRB and Non-FRB were analysed using conventional methods for food analysis.

### Measurement of total phenolic content

The total phenolic contents of FRB and Non-FRB were determined colourimetrically using Folin-Ciocalteu reagent according to the procedure specified by Ainsworth [23] but with slight modifications. Three grams of the fresh samples were mixed with 30 ml of 70 % methanol. The mixtures were vortex for 30 min. The mixtures were then centrifuged at 1400 × *g* at 4 °C for 15 min and the supernatants were collected. Repeated extraction was done for 45 min and 90 min vortex. The supernatants were then pooled for analysis.

To measure the total phenolic content, 0.1-ml aliquots of the extracts were mixed with 0.75 ml of 10-fold diluted Folin-Ciocalteu reagent and allowed to stand at 25 °C for 10 min. Next, 0.75 ml of 2 % Na_2_CO_3_ was added to each mixture and the solutions were allowed to stand in the dark for 45 min at 25 °C. Absorbance was then measured at 765 nm using a spectrophotometer. Standard curves were plotted using standard catechin and gallic acid solutions (Sigma-Aldrich Co., St. Louis, MO, USA). Total phenolic content was expressed as the gallic acid equivalents per gram of each sample of dried rice bran.

### Animals

Male SHRSP/Izm rats were obtained from Japan SLC (Hamamatsu, Japan) and used in the study. The rats were kept in two-chambered individual stainless steel cages in a controlled atmosphere (temperature: 23 ± 3 °C; humidity: 50 ± 10 %; and a 12/12 h light–dark cycle). The rats were given free access to AIN-93M standard rat diet and drinking water for 1 week before the experiments. The animal use and care protocols were reviewed and approved by the Animal Research-Animal Care Committee of Tohoku University (Sendai, Japan).

### Biochemical analyses

The serum levels of glucose, total cholesterol (TC), triglycerides (TG), and high-density lipoprotein cholesterol (HDL-C) were measured by enzymatic colorimetric methods (Wako Pure Chemical Industries, Ltd.) according to the manufacturer’s instructions. Serum levels of low-density lipoprotein cholesterol (LDL-C) were calculated using the Friedewald formula [(TC – HDL-C) – (1/5 × TG)]. Plasma adiponectin levels were measured using a rat adiponectin ELISA kit (Otsuka Pharmaceutical Co., Ltd., Tokyo, Japan), whereas serum leptin levels were measured using a rat leptin ELISA kit (Yanaihara Institute Inc., Fujinomiya, Japan). Liver TC and TG levels were measured with the same kits used for measuring the serum TC and TG levels, following the extraction of liver samples with methanol/chloroform (1:2, *v/v*).

### Single-dose oral administration study

Twelve 11-week-old male SHRSP/Izm rats were used in this study. After the 1-week acclimatization period, the rats were divided into 3 groups of 4 rats each and administered FRB, Non-FRB, or water (control). FRB and Non-FRB were administered at 2 g/kg body weight. Each rat was deprived of food and water for 16 h before dose administration. The body weight and initial systolic BP of each rat were measured before and at 1, 2, 4, and 6 h after dose administration. Six BP measurements were taken for each rat. All the BP measurements in this study were taken by the same person.

### Chronic supplementation study

Eighteen, 9-week-old, male, body-weight-matched SHRSP/Izm rats were used in this study. After the 1-week acclimatization period, the rats were divided into 3 groups of 6 rats each and supplied 5 % of FRB, Non-FRB, or water (control) (Table 1) for 4 consecutive weeks. Food intake and body weight were measured every other day and weekly, respectively. Systolic, diastolic, and mean arterial blood pressure were measured weekly.

**Table 1:** Composition of experimental diets

Ingredient (g/kg)	Control	Non-FRB	FRB
Corn starch	602.65	589.65	589.65
Casein	140.5	133	133
Sucrose	95	95	95
Cellulose	58.5	47.5	47.5
Soybean oil	43	38	38
Mineral mixture	38.25	33.25	33.25
Vitamin mixture	9.5	9.5	9.5
Choline bitartrate	2.37	2.375	2.375
L-Cystine	1.71	1.71	1.71
*tert*-Butylhydroquinone	0.0076	0.0076	0.0076
Test material	8.5 (water)	50.0	50.0
Total	1000	1000	1000

*FRB* fermented rice bran, *Non-FRB* non-fermented rice bran

After the 4 weeks of chronic supplementation with the various treatments, each rat was sacrificed by direct decapitation. Blood was collected and allowed to coagulate for 30 min, after which serum was obtained by centrifuging the coagulated blood at 1400 × *g* for 15 min. The serum samples were stored at −20 °C for measurements later. The livers were collected and dissected immediately after sacrifice, washed with normal saline, frozen in liquid nitrogen, and stored at −80 °C until analysis.

#### Oral glucose tolerance test (OGTT) and insulin tolerance test (ITT)

The OGTT was conducted when the rats were 13 weeks old. The test was done after fasting the rats for 16 h. Blood samples were collected from the tail vein before and at 30, 60, and 120 min after the oral administration of glucose (1.8 g/kg body weight) to each rat via a gastric tube. The levels of glucose and insulin in plasma were then analysed. Area under the curve (AUC) was calculated based on a previously published method [24].

The animals were subjected to the ITT 3 days before they were sacrificed and after a 5-h fast. Blood samples were collected from the tail vein before and at 30, 60, and 120 min after the rats were administered human insulin (Humulin R 100 IU/ml; Eli Lilly and Company, Indianapolis, IN, USA) intraperitoneally (0.6 U/kg body weight). Glucose levels in the plasma samples were then measured.

### Measurement of ACE inhibitory activity

The ACE inhibitory activities of FRB and Non-FRB were determined according to a previously published method [25]. Briefly, enzyme and indicator working solutions were prepared according to the manufacturer’s instructions. Aliquots of serum (20 μl) were placed in the wells of a 96-well microplate. Next, 20 μl and 40 μl of deionized water were added for the ‘blank1’ and ‘blank2’ wells, respectively. Afterwards, 20 μl of substrate buffer was added to each well, followed by the addition of 20 μl of enzyme working solution to the experimental wells and blank1. The plate was then incubated at 37 °C for 1 h. After incubation, 200 μl of indicator working solution was added to each well and the plate was incubated again at 25 °C for 5 min. Absorbance was measured at 450 nm using a spectral microplate auto-reader. ACE inhibitory activity was calculated using the following equation:

$$ \mathrm{ACE}\kern0.5em \mathrm{inhibitory}\kern0.5em \mathrm{activity}\left(\%\kern0.5em \mathrm{Inhibition}\kern0.5em \mathrm{rate}\right)=\left({\mathrm{A}}_{\mathrm{blank}1}\hbox{--} {\mathrm{A}}_{\mathrm{sample}}\right)/\left({\mathrm{A}}_{\mathrm{blank}1}\hbox{--} {\mathrm{A}}_{\mathrm{blank}2}\right)\times 100 $$

### RNA preparation and quantitative reverse transcription polymerase chain reaction (RT-PCR)

Total RNA was isolated from the liver tissues of the rats using a guanidine isothiocyanate-based reagent (Isogen; Nippon Gene, Tokyo, Japan) according to the manufacturer’s instructions. After isolating the total RNA, the ratio at wavelength of 260 and 280 nm was measured. Agarose gel electrophoresis was performed to expedite the quantitative and qualitative analyses of the isolated RNA. Four micrograms of RNA was used as a template to synthesize cDNA as previously described [21]. Aliquots of the obtained cDNA were then used as templates for the subsequent quantitative PCR using an Applied Bioscience (Foster City, CA, USA) 7300 real-time PCR system. The relative gene expression levels were normalized to the amount of eukaryotic elongation factor-1α1 mRNA. The genes were amplified using cDNA specific primers. The following primers were used: glucose-6-phosphatase catalytic subunit (G6PC): TTGTGCATTTGCTAGGAAGAGAAG (forward) TCTAAAGACCCAGGCATAACTGAAG (reverse); phosphoenolpyruvate carboxykinase (PEPCK): GAGGACATTGCCTGGATGAAGTTT (forward) TGGGTTGATGGCCCTTAAGT (reverse); fatty acid synthase (FASN): GGCTCACACACCTACGTATTGG (forward) TGCTTAATGAAGAAGCATATGGCTT (reverse); acetyl-CoA carboxylase (ACC): TTGTGGAAGTGGAAGGCACAG (forward) CCTTATTATTGTCCCAGACGTAAGC (reverse); stearoyl-CoA desaturase-1 (SCD1): CTGGAGATGGGAGCCACAAG (forward) CAGGAACTCAGAAGCCCAGAA (reverse); 3-hydroxy-3-methylglutaryl-CoA reductase (HMGCR): AATTGTGTGTGGCACTGTGATG (forward) GATCTGTTGTGAACCATGTGACTTCT (reverse); sterol regulatory element-binding protein 1c (SREBP-1c): GGAGCCATGGATTGCACATT (forward) GCTTCCAGAGAGGAGCCCAG (reverse); Carbohydrate response element binding protein alpha (ChREBPα): CGACACTCACCCACCTCTTC (forward) TTGTTCAGCCGGATCTTGTC (reverse); liver X receptor alpha (LXRα): ACCTCTGCGATCG AGGTGAT (forward) AAAGTCTTCCGGGTTGTAACTGA (reverse); and elongation factor-1α1: GATGGCCCCAAATTCTTGAAG (forward) GGACCATGTCAACAATTGCAG (reverse).

### Western blot analysis

The rat livers were homogenized in phosphate-buffered saline containing protease (cOmplete protease inhibitor cocktail; Roche Applied Science, Mannheim, Germany) and phosphatase (PhosSTOP phosphatase inhibitor cocktail, Roche Applied Science) inhibitors. The protein concentrations in the lysates were measured using a protein assay reagent (Bio-Rad, Hercules, CA, USA). Afterwards, 7.5 μg of protein in sodium dodecyl sulfate (SDS) gel loading buffer was resolved on a 10–20 % SDS-polyacrylamide gel. The separated proteins were transferred onto Immobilon-P membrane (Millipore, Billerica, MA, USA). The membrane was incubated with a blocking buffer (10 mmol/l Tris–HCl at pH 7.4, 150 mmol/l HCl, 0.1 % Tween 20, and 5 % bovine serum albumin) for 1 h. Next, the membrane was incubated overnight with the blocking buffer containing antibodies against AMP-activated protein kinase (AMPK)-α1 (Millipore) at a dilution of 1:10,000 and antibodies against p-AMPKα (Thr 172; Millipore) at a dilution of 1:5000. Immobilon western detection reagent (Millipore) was used with a luminescent image analyser (LAS-4000 mini; Fujifilm, Tokyo, Japan). The relative expression levels of each protein were normalized according to the amount of α-tubulin (Sigma-Aldrich Co.) detected by the antibody against the protein.

### Statistical analysis

Data are represented as mean ± standard error of the mean (SEM). Statistical analysis was performed by one-way analysis of variance followed by Tukey’s multiple comparison test. The analysis was performed using SigmaPlot software version 12.5 (San Jose, CA, USA). A *P* value < 0.05 was considered statistically significant.

## Results

### Macronutrients in FRB and Non-FRB

The dual fermentation process improved on the characteristic flavour and macronutrient composition of the rice bran (Table 2). The results show that the lipid, dietary fibre, and total phenolic contents of FRB are higher than those of non-FRB. These indicate that the fermentation process enhanced the natural flavour and the chemical and secondary metabolite content of the rice bran.

**Table 2:** Analysis of macronutrients in fermented and non-fermented rice bran

Parameters (g/100 g of rice bran)	Non-FRB	FRB
Water	6.7	16.9
Protein	14.4	15.4
Lipid	6.7	10.0
Dietary fibre	4.4	22.0
Ash	8.8	10.0
Carbohydrate	49.3	13.1
Total phenolic content	6.5	8.6

Total phenolic content was calculated as the gallic acid equivalent per gram of dried rice bran. *FRB* fermented rice bran, *Non-FRB* non-fermented rice bran

### Effect of FRB after a single-dose oral administration

**Fig. 1:**
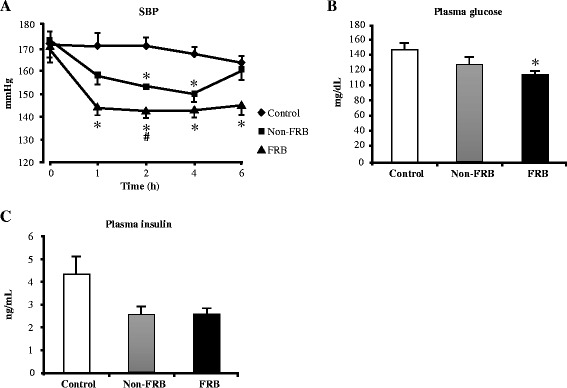
Effects of a single-dose oral administration of FRB in SHRSP. (**a**) Systolic blood pressure (SBP), (**b**) plasma glucose, and (**c**) insulin levels 2 h after FRB administration. All data are presented as mean ± SEM (*n* = 4). ^*^, ^#^ *P* < 0.05 compared to control, Non-FRB. *FRB* fermented rice bran, *Non-FRB* non-fermented rice bran, *SEM* standard error of the mean, *SHRSP* stroke-prone spontaneously hypertensive rats

Rats that had systolic BP of approximately 175 mm/Hg were used to investigate whether or not ingesting FRB at a single oral dose of 2 g/kg body weight produces an antihypertensive effect (Fig. 1a). The mean systolic BP of the control group was almost constant for 6 h and slightly decreased afterwards. A BP lowering effect was observed at 1, 2, and 4 h after the administrations of FRB and Non-FRB to the SHRSP. However, after 6 h, BP lowering was constant in the FRB group, and similar in the Non-FRB and control groups. The maximum reduction in BP observed was approximately 35 mmHg and it occurred in the FRB group from 1 to 4 h after dose administration. The effects of the single oral dose of FRB on blood glucose and insulin levels are shown in Fig. 1b and c, respectively. Two hours after administering the single dose FRB, there was a reduction in plasma glucose than that in the control group. In addition, plasma insulin levels tended to decrease in both the FRB and Non-FRB groups. However plasma TC and TG levels were the same values in all the groups (data not shown).

### Antihypertensive effect of FRB after chronic supplementation

**Fig. 2:**
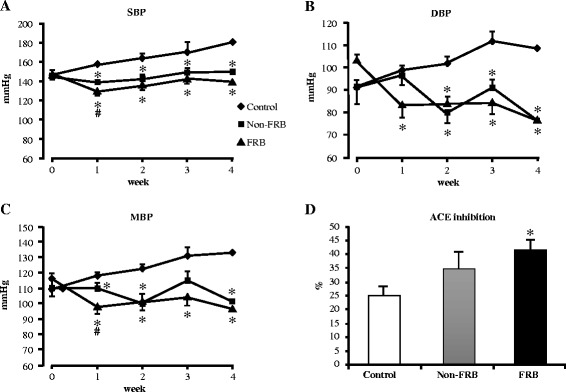
Effects of chronic supplementation with FRB on blood pressure in SHRSP. (**a**) Systolic blood pressure (SBP), (**b**) diastolic blood pressure (DBP), (**c**) mean blood pressure (MBP), and (**d**) serum angiotensin-converting enzyme (ACE) inhibitory activity after 4 weeks of FRB supplementation. All data are presented as mean ± SEM (*n* = 6). ^*^, ^#^ *P* < 0.05 compared to control, Non-FRB. *FRB* fermented rice bran, *Non-FRB* non-fermented rice bran, *SEM* standard error of the mean, *SHRSP* stroke-prone spontaneously hypertensive rats

Chronic supplementation with either FRB or Non-FRB decreased BP elevation of the rats (Fig. 2a, b, and c). Systolic, diastolic, and mean BP significantly decreased throughout the experiment in the FRB group but BP elevation remained constant in the control group. Non-FRB reduced diastolic BP from the second week of the experiment; however, reduction in diastolic BP was consistent in the FRB group throughout the experiment. At the end of the experiment, the mean systolic BP was 181 ± 3.6, 149 ± 4.3, and 138 ± 4.4 mmHg for the control, Non-FRB, and FRB groups, respectively. Furthermore, serum ACE inhibitory activity was significantly higher in the FRB group than in the control group whereas Non-FRB group had no significant difference (Fig. 2d). The results obtained indicate that FRB has a hypotensive effect.

### Effect of chronic supplementation with FRB on growth, liver, and serum parameters in SHRSP

**Fig. 3:**
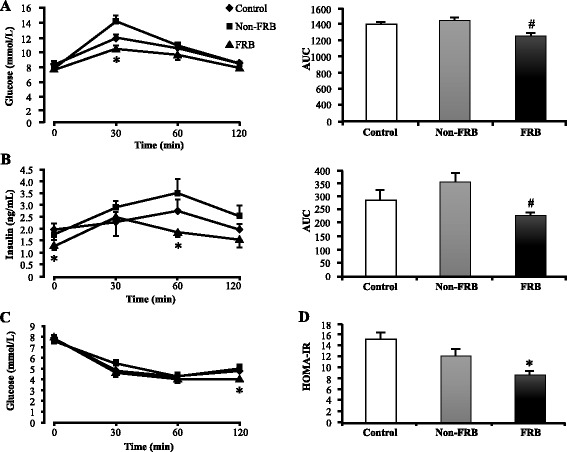
Effects of FRB on plasma glucose and insulin levels. **a** Plasma glucose levels after oral glucose tolerance test (OGTT) (*left*) and the resulting area under the curve (*right*). **b** Plasma insulin levels after OGTT (*left*) and the resulting area under the curve (*right*). **c** Plasma glucose levels after insulin tolerance test (ITT). **d** Homeostatic model assessment of insulin resistance (HOMA-IR) index. Values are expressed as mean ± SEM (*n* = 6). ^*^, ^#^ *P* < 0.05 compared to control, Non-FRB. *FRB* fermented rice bran, *Non-FRB* non-fermented rice bran, *SEM* standard error of the mean

Chronic supplementation with FRB significantly reduced food intake, body weight, and epididymal fat mass (Table 3). No differences were observed in the kidney and heart weights; however, liver weight showed a tendency to reduce (data not shown). FRB significantly improved glucose tolerance as well as the AUC of glucose and insulin (Fig. 3a and b). In addition, FRB improved insulin resistance by reducing the insulin resistant index, HOMA-IR (homeostatic model assessment of insulin resistance), as well as glucose levels after ITT (Fig. 3c and d). Table 3 summarizes the serum, liver, and growth parameters measured in this study. After 4 weeks of chronic supplementation with FRB, plasma levels of glucose and insulin reduced. At the end of the study, FRB had significantly lowered hepatic TG and TC levels. In addition, serum TG and TC levels were lower in the FRB group than in the control group. Supplementation with FRB caused a significant increase in serum adiponectin (both high molecular weight and total adiponectin) levels and a significant decrease in serum fasting leptin levels. Moreover, FRB supplementation increased leptin sensitivity and subsequently decreased the leptin/adiponectin ratio.

**Table 3:** Effects of fermented and non-fermented rice bran on growth, serum, and liver parameters

Parameters	Control	Non-FRB	FRB
Growth			
Cumulative food intake (g)	398.18 ± 14.73	353.49 ± 32.05	311.63 ± 11.33^*^
Body weight gain (g)	81.21 ± 2.17	77.95 ± 2.91	71.18 ± 3.16^*#^
Epididymal fat mass (g/100 g BW)	1.99 ± 0.03	2.10 ± 0.04	1.72 ± 0.08^*#^
Serum			
Glucose (mmol/l)	11.17 ± 0.95	7.99 ± 0.40^*^	8.31 ± 0.32^*^
Insulin (ng/ml)	4.49 ± 0.35	3.42 ± 0.43	2.28 ± 0.19^*^
AST (IU/l)	187.33 ± 6.96	231.50 ± 16.40^*^	189.33 ± 8.04^#^
TC (mg/dl)	80.00 ± 5.50	74.00 ± 2.03	76.17 ± 2.49
TG (mg/dl)	87.50 ± 3.33	89.33 ± 9.20	73.50 ± 5.94
LDL-C (mg/dl)	33.83 ± 4.45	27.63 ± 1.07	32.63 ± 2.14
HDL-C (mg/dl)	28.67 ± 1.33	28.50 ± 0.62	28.83 ± 0.87
Non-fasting leptin (ng/ml)	1.35 ± 0.10	1.88 ± 0.15	3.46 ± 2.60
Fasting leptin (ng/ml)	0.95 ± 0.08	0.78 ± 0.07	0.52 ± 0.05^*^
Leptin sensitivity (%)	28.35 ± 6.22	51.48 ± 6.75	59.55 ± 9.52^*^
High molecular adiponectin (μg/ml)	0.85 ± 0.04	1.01 ± 0.06	1.01 ± 0.03^*^
Total adiponectin (μg/ml)	8.70 ± 0.15	8.24 ± 0.14	9.092 ± 0.12^#^
Leptin/adiponectin ratio	0.11 ± 0.01	0.09 ± 0.01	0.05 ± 0.01^*^
Liver			
Liver TG (mg/g tissue)	24.05 ± 0.78	23.95 ± 3.44	13.75 ± 2.09^*^
Liver TC (mg/g tissue)	3.25 ± 0.21	2.92 ± 0.31	1.61 ± 0.22^*^
Total lipid (mg/g tissue)	82.71 ± 18.26	78.99 ± 6.14	60.69 ± 11.47

Values are presented as means ± SEM, (*n* = 6). ^*^, ^#^ *P* < 0.05 compared to control, Non-FRB. *AST* aspartate aminotransferase, *BW* body weight, *FRB* fermented rice bran, *HDL-C* high-density lipoprotein cholesterol, *LDL-C* low-density lipoprotein cholesterol, *Non-FRB* non-fermented rice bran, *TC* total cholesterol, *TG* triglycerides

### Effect of FRB on hepatic gene expression and AMPK activation

**Fig. 4:**
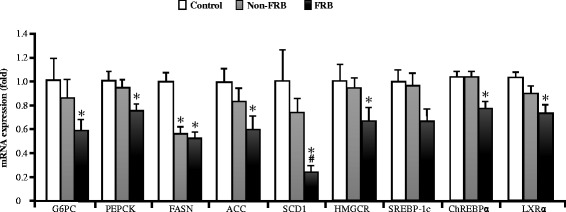
Effects of FRB on hepatic gene expression. *ACC* acetyl-CoA carboxylase, *ChREBPα* carbohydrate-responsive element-binding protein alpha, *FASN* fatty acid synthase, *G6PC* glucose-6-phosphatase catalytic subunit, *HMGCR* 3-hydroxy-3-methylglutaryl-CoA reductase, *LXRα* liver X receptor alpha, *PEPCK* phosphoenolpyruvate carboxykinase, *SCD1* steroyl-CoA desaturase-1, *SREBP-1c* sterol regulatory element-binding protein 1c. Values are expressed as mean ± SEM (*n* = 6). ^*^, ^#^ *P* < 0.05 compared to control, Non-FRB. *FRB* fermented rice bran, *Non-FRB* non-fermented rice bran, *SEM* standard error of the mean

**Fig. 5:**
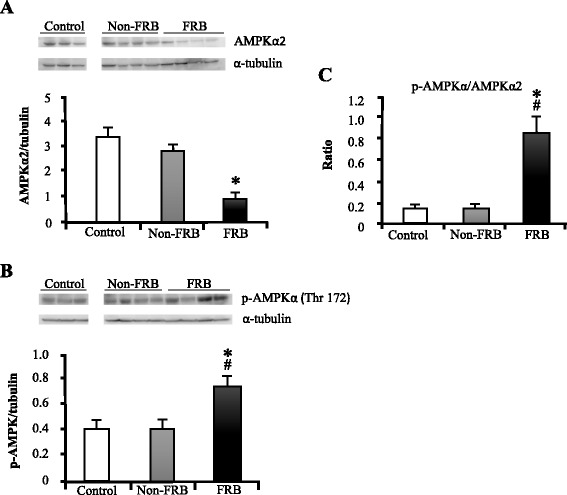
Effects of FRB on AMPKα2 and p-AMPKα levels. (**a**) AMPKα2 and (**b**) p-AMPKα protein levels from quantitative western blot analysis. **c** p-AMPKα/AMPKα2 ratio. Data were normalized with α-tubulin. Values are expressed as mean ± SEM (*n* = 3–4). ^*^, ^#^ *P* < 0.05 compared to control, Non-FRB. *AMPK* AMP-activated protein kinase, *FRB* fermented rice bran, *Non-FRB* non-fermented rice bran, *SEM* standard error of the mean

The mRNA expression levels of LXRα, SREBP-1c, and ChREBPα in the liver were measured to clarify how FRB ameliorates metabolic syndrome in SHRSP. After chronic supplementation with FRB, the relative mRNA expression levels of the aforementioned transcription factors and their target genes such as G6PC, PEPCK, FASN, ACC, SCD1, and HMGCR were suppressed (Fig. 4). Moreover, quantitative western blot analysis showed that FRB significantly increased p-AMPK/AMPK ratio as well as phosphorylation of p-AMPKα in the liver (Fig. 5). Thus, FRB may activate the AMPK signalling pathway in the liver.

## Discussion

This study is the first to demonstrate that dual fermentation of rice bran enriches the functional ingredients in rice bran. FRB therefore contains higher levels of lipid, dietary fibre, and phenolic compounds more than Non-FRB does (Table 2). The results obtained from this study indicate that FRB improves hypertension, insulin resistance, glucose impairment, serum adiponectin level, and AMPK activation in SHRSP. Thus, FRB may be a potent functional food that can be used for the management of metabolic syndrome.

Adiponectin is an important adipokine for insulin sensitization and is involved in some homeostatic functions such as the regulation of glucose and lipid metabolism [26]. A recent study showed that antihypertensive drugs could improve the sensitivities of adiponectin and leptin in SHRSP [27]. In the present study, FRB improved adiponectin and leptin impairments in SHRSP, which results in an improvement in glucose and lipid metabolism. Plasma adiponectin levels are inversely related to adiposity and directly associated with leptin sensitivity [28]. Thus, it appears that FRB exerted its action on leptin sensitivity and body fat mass via the stimulation of adiponectin secretion even though the exact mechanisms involved are still unknown.

Phenolic compounds and dietary fibre are used in formulating food products to improve the functionalities and health benefits of such products. Phenolic compounds and dietary fibre produce health benefits by reducing cholesterolemia, modifying glycemic responses, and preventing the development of cardiovascular diseases [29, 30]. Microorganisms are used in the brewing and food industries to produce fermented products. They are also used to produce aroma compounds and secondary metabolites for use in processed foods. Our results confirm that FRB is a functional food that can be effective in the management of metabolic syndrome and for the prevention of lifestyle-related diseases.

We observed that after administration, a single oral dose of FRB (2 g/kg body weight) lowers BP within 6 h. We also found that FRB has the capacity to decrease plasma glucose and insulin levels (Fig. 1). Chronic supplementation with 5 % FRB for 4 weeks increased serum ACE inhibitory activity (Fig. 2). This corroborates the results in a previous report, which indicated that fractions of enzyme-treated rice bran improved BP elevation in SHRSP via the inhibition of ACE activity [21]. Furthermore, FRB supplementation enhanced serum adiponectin levels (both high molecular weight and total adiponectin) and improved serum and liver lipid profiles (Table 3). FRB supplementation also improved glucose tolerance and insulin resistance (Fig. 3). It was shown in a previous study that L-tryptophan, which is one of the identified functional ingredients in enzyme-treated rice bran, improved blood pressure as well as blood glucose and insulin levels [31]. It was observed that the L-tryptophan content of FRB (30 mg/100 g) is much higher than that of Non-FRB (4 mg/100 g). In the liver, there is *de novo* glucose synthesis mainly from lactate, alanine, pyruvate, and glycerol. In the present study, FRB reduced the mRNA levels of enzymes involved in gluconeogenesis (PEPCK and G6PC), which are the rate limiting enzymes in gluconeogenesis. Insulin can inhibit the transcriptional activity of forkhead box protein O1, which regulates the transcription of PEPCK. The aforementioned mechanisms were therefore involved in the improvements in serum glucose and insulin levels by FRB in this study. Studies have shown that brown rice bran and enzyme-treated rice bran improve glucose tolerance and insulin resistance in mouse and rat models [21, 32]. Altogether, our results suggest that FRB regulates glucose and lipid metabolism and contributes effectively to improving hypertension in SHRSP better than Non-FRB does.

Moreover, we observed that LXRα, SREBP-1c, and ChREBPα mRNA expression levels were downregulated after FRB supplementation in the SHRSP. Glucose and insulin coordinate hepatic lipogenesis and the glycolytic gene expression [33]. Both glucose and insulin are potent factors that are involved in inducing the transcription of key enzyme genes in glycolysis and *de novo* lipogenesis [34]. It has been found that rice bran derivatives reduce plasma and liver lipids, whereas protein hydrolysates from rice bran improve insulin and leptin sensitivity in high-fat diet-induced metabolic syndrome in hamsters [6, 14]. Insulin triggers the transcriptions of glycolytic and lipogenic enzymes by activating SREBP-1c and LXRα [35, 36]. Therefore, chronic supplementation with FRB downregulated the mRNA expression levels of LXRα, SREBP-1c, and ChREBPα, as well as their target genes such as G6PC, PEPCK, HMGCR, FASN, SCD1, and ACC in the liver (Fig. 4). Thus, the decreased serum glucose and liver TG levels due to the intake of FRB may have been caused by an enhanced serum level of adiponectin, which regulates glucose and lipid metabolism.

FRB increased the activation of AMPK in the liver (Fig. 5) and quantitative western blot analysis showed that FRB increased the phosphorylation of AMPKα. This indicates that FRB may activate the adiponectin/AMPK signalling pathway. A study in humans has shown that plasma adiponectin concentration negatively correlates with lipid metabolism [37]. However, a previous study demonstrated that the phosphorylation and activation of AMPK are stimulated by adiponectin in the liver. The study also indicated that adiponectin improved insulin sensitivity and decreased plasma glucose levels via the AMPK-peroxisome proliferator-activated receptor gamma coactivator 1-alpha signalling pathway in the liver [38].

The results of the current study verified that the intake of FRB could increase plasma adiponectin levels. This results in an activation of AMPK and a downregulation of the mRNA expression of genes involved in gluconeogenesis and lipogenesis in the liver. This suggests that enhancing serum adiponectin levels and downregulating transcription factors involved in hepatic glucose and fat metabolism may be a promising strategy to prevent metabolic-related diseases characterized by insulin resistance and hyperlipidemia. Therefore, dietary supplementation with FRB can mitigate metabolic syndrome.

## Conclusions

Our results clearly indicate that dual fermentation of rice bran with *Aspergillus kawachii* and lactic acid bacteria enriches the functional value of rice bran. This is because metabolic syndrome was better alleviated with FRB supplementation than with Non-FRB supplementation. FRB effectively reduced BP by increasing serum ACE inhibitory activity. FRB also increased serum adiponectin levels and improved glucose and lipid metabolism. We therefore suggest FRB as an effective functional food to reduce the risks of lifestyle-related diseases. Further studies are however needed to elucidate the complete physiological effect of FRB and confirm its detailed mechanism of action at the molecular level.

## Abbreviations

ACE: Angiotensin-converting enzyme
FRB: Fermented rice bran
SHRSP: Stroke-prone spontaneously hypertensive rat
